# Endovascular treatment of acute ischemic stroke due to tandem lesions of the anterior cerebral circulation: a multicentric Italian observational study

**DOI:** 10.1007/s11547-020-01331-7

**Published:** 2021-01-27

**Authors:** Sandra Bracco, Matteo Zanoni, Tommaso Casseri, Davide Castellano, Samuele Cioni, Ignazio Maria Vallone, Paola Gennari, Maria Antonietta Mazzei, Daniele Giuseppe Romano, Mariangela Piano, Chiara Comelli, Rossana Tassi, Elisa Francesca Maria Ciceri

**Affiliations:** 1 Unit of Interventional Neuroradiology, Department of Neurology and Human Movement Sciences, Azienda Ospedaliera Universitaria Senese, Siena, Italy; 2 Unit of Diagnostic Imaging, Department of Medical, Surgical and Neuro Sciences, University of Siena, Azienda Ospedaliera Universitaria Senese, Santa Maria Alle Scotte Hospital, Viale M. Bracci 16, 53100 Siena, Italy; 3 Neuroradiology Department, Azienda Ospedaliera Universitaria Integrata Verona, Verona, Italy; 4 Neuroradiology Department, Azienda Socio Sanitaria Territoriale Grande Ospedale Metropolitano Niguarda, Milan, Italy; 5 Department of Interventional Radiology and Neuroradiology, S. Giovanni Bosco Hospital, Azienda Sanitaria Locale Città di Torino, Turin, Italy; 6 Neuroradiology Unit, Azienda Ospedaliera Universitaria San Giovanni di Dio e Ruggi d’Aragona, Salerno, Italy; 7 Stroke Unit, Azienda Ospedaliera Universitaria Senese, Siena, Italy

**Keywords:** Acute ischemic stroke, Endovascular therapy, Stroke mechanical thrombectomy, Carotid plaque, Carotid dissection, Carotid stenting

## Abstract

**Purpose:**

Acute ischemic stroke (AIS) due to tandem lesions (TLs) of extracranial Internal Carotid Artery and Anterior Cerebral Circulation are challenging for endovascular treatment (EVT). This study aims to evaluate feasibility, safety and efficacy of EVT for TLs’ AIS, with or without emergent carotid artery stenting (eCAS), in a real-life scenario.

**Methods:**

Retrospective review of prospectively collected non-randomized thrombectomy databases from five stroke centers between 2015 and 2019. Consecutive patients with TLs’ AIS were selected. Clinical, neuroimage and procedure features, as well as antiplatelet therapy regimen, were evaluated. Primary outcome was 90-day mRS ≤ 2. Secondary outcomes included: mTICI score 2b-3, extracranial recanalization, procedural complications, symptomatic intracerebral hemorrhage (SICH) and 90-day mortality.

**Results:**

Two hundred twenty-seven patients were enrolled (67.8% males; mean age 65.9 ± 12.9 years). We obtained mTICI 2b-3 in 77.1%, extracranial recanalization in 86.8%, 90-day mRS (available in 201/227 cases) ≤ 2 in 49.8%. Procedural complications occurred in 16.7%, SICH in 9.7%; 90-day mortality rate (available in 201/227 cases) was 14.4%. The strongest predictors of good clinical outcome were young age (*p* < 0.0001), low baseline NIHSS (*p* = 0.008), high baseline ASPECTS (*p* < 0.0001), good collateral flow (*p* = 0.013) and extracranial recanalization (*p* = 0.001). The most significant predictors of SICH were low baseline ASPECTS (*p* < 0.0001), occurrence of complications (*p* < 0.0001) and eCAS (*p* = 0.002).

**Conclusion:**

In our real-life series, the EVT for TLs’ AIS was feasible, safe and effective in improving 90-day functional outcome with acceptable morbi-mortality rates. ECAS increased the risk of SICH, independently from the antiplatelet therapy regimen.

## Introduction

Since 2015, many trials [1–5] assessed the superiority of mechanical thrombectomy in the treatment of acute ischemic stroke (AIS) in comparison with standard intravenous fibrinolysis (IV-FL). The same cannot be said for tandem lesions (TLs) of extracranial Internal Carotid Artery (ICA) and Anterior Cerebral Circulation [6], representing about 15% of AIS [7].

TLs consist in the presence of occlusion, sub-occlusion or stenosis of extracranial ICA, due to dissection or atheromatous plaque, together with simultaneous intracranial large vessel occlusion [7–11].

They show both poor responsiveness to IV-FL [12] and technical difficulties for endovascular treatment (EVT), resulting in low recanalization rates and more unfavorable outcomes [10]. Moreover, there is a lack of current guidelines about their management [13].

The aim of this study is to assess feasibility, safety and efficacy of EVT for AIS due to TLs. A comprehensive overview of these complex endovascular situations will also be provided, particularly focusing on the causal link between extracranial and intracranial lesions which constitute TLs.

## Methods

### Patient selection, variables and outcomes

This retrospective study was approved by the institutional review board at our hospital. The requirement for written informed consent was waived because of the retrospective study design. Prospectively collected non-randomized thrombectomy databases from five major Italian stroke centers were retrospectively reviewed. All the consecutive patients with anterior circulation AIS due to TLs who underwent EVT between 01 January 2015 and 31 August 2019 were enrolled.

Angiography inclusion criteria were:

1. Extracranial ICA occlusion or sub-occlusion, due to dissection or atheromatous plaque, and simultaneous intracranial vessel occlusion (IVO);
2. Extracranial ICA stenosis, due to unstable plaque, ≥ 80% NASCET (North American Symptomatic Carotid Endarterectomy Trial) [14] and simultaneous IVO;
3. Extracranial ICA occlusion or sub-occlusion without IVO but with insufficient Willis compensation (defined as “hemodynamic TLs” by the Authors).

Exclusion criteria were:

1. Absence of TLs;
2. Extracranial ICA occlusion or sub-occlusion and simultaneous IVO by another cause, e.g., cardiogenic embolism;
3. Extracranial ICA stenosis, due to stable plaque, ≤ 80% NASCET and simultaneous IVO, being the two lesions not apparently linked;
4. Pre-stroke modified Rankin Scale (mRS) score > 2;
5. Age < 18 years.

Patients’ relevant data were collected. Clinical features included: gender, age, risk factors (hypertension, diabetes mellitus, dyslipidemia and smoking habit), baseline National Institutes of Health Stroke Scale (NIHSS) and administration of IV-FL. Neuroimaging features were: baseline Alberta Stroke Program Early CT Score (ASPECTS), intracranial lesion site (ICA not T-type, ICA T-type, M1-Middle Cerebral Artery [MCA], M2 or distal-MCA, “hemodynamic TLs”), etiology (plaque or dissection) and type (occlusion, sub-occlusion or stenosis) of extracranial lesion and collateral flow status, assessed with the ASITN/SIR (American Society of Interventional and Therapeutic Neuroradiology/Society of Interventional Radiology) grading system [15] on Digital Subtraction Angiography (DSA) and subsequently classified as "good" (ASITN/SIR 3–4), "moderate" (ASITN/SIR 2) or "poor" (ASITN/SIR 0–1).

Procedural features included: type of anesthesia (general anesthesia or conscious sedation, according to local guidelines), intracranial thrombectomy technique (Thromboaspiration Alone [TA] or Stent-retriever Combined Thrombectomy [SCoT]), emergent carotid artery stenting (eCAS) performed in anterograde (i.e., treatment of the proximal lesion first) or retrograde (i.e., treatment of the distal lesion first) fashion, super selective administration of adjunct antiplatelet therapy during the procedure (Acetylsalicylic Acid [ASA], P2Y12 inhibitors or Glycoprotein IIb/IIIa [GpIIb/IIIa] inhibitors), time from Onset To Groin puncture (OTG) and from Groin puncture To Reperfusion (GTR).

After the procedure, all the patients were admitted to a neuro-dedicated Intensive Care Unit or to a Stroke Unit, under a strict blood pressure control.

The primary outcome was functional independence (mRS ≤ 2) at 90-day follow-up. Secondary outcomes were good intracranial reperfusion (modified Thrombolysis In Cerebral Infarction [mTICI] score 2b-3) and extracranial ICA recanalization.

Data regarding complications, such as embolization of new territories and iatrogenic dissection or perforation (recognized at the time of treatment) and symptomatic intracerebral hemorrhage (SICH), were also collected. SICH was defined, according to the SWIFT PRIME trial [4], as any parenchymal hematoma, intracerebral, subarachnoid or intraventricular hemorrhage causing a decline of four or more points in the NIHSS within 24 h from the end of the revascularization procedure.

Neuroimaging data (CT and DSA) were consensually reviewed by an experienced interventional neuroradiologist and a senior radiology resident with experience in interventional neuroradiology.

### Statistical analysis

Patients data were collected in a single database. All data were validated and then submitted to usual descriptive techniques. Continuous variables were described either as mean and standard deviation (SD) or as median and interquartile range (IQR), depending on their distribution, which was checked using the Shapiro–Wilk test. Categorical variables (nominal and ordinal) were provided as relative and absolute frequency tables.

Comparisons between continuous variables were performed using either the Student’s t test or the Mann–Whitney U test, depending on their distribution and on the variance homogeneity (previously evaluated through the Levene’s test). The Fisher’s exact test was used for comparison between categorical variables.

The strongest predictors of 90-day functional independence and of SICH were identified using the multivariate logistic regression with the likelihood ratio test for the model as a whole and through the Wald’s test for each regressor. At the beginning, the multivariable regression included the regressors having *p* < 0.2 at univariate analysis, which were subsequently excluded in a stepwise fashion, in order to get the best BIC and McFadden R^2^ values.

Statistical analyses were performed using Stata/SE 12.0 (The Statacorp, College Station, USA). All calculated *p*-values were two-sided, and statistical significance was assumed for *p* < 0.05.

## Results

During the study period, 1482 patients with anterior circulation AIS underwent EVT, with 227 of them having a TL. Over the 227 patients, 26 were lost at 90-day follow-up and therefore 90-day mRS and mortality were available for 201/227 (88.6%) patients, while intra-procedural and post-procedural data were available for all 227 patients.

Clinical, neuroimage and procedure data are shown in Table 1, while the results of statistical analysis for functional independence (mRS ≤ 2), technical outcomes (mTICI 2b-3 and extracranial ICA recanalization) and SICH are reported in Table 2, 3 and 4, respectively.

**Table 1:** Clinical, Neuroimaging and Procedure Variables and Outcomes

Clinical variables		Procedure variables	
Age-years, mean (SD)		General Anesthesia, n (%)	104 (45.8)
All	65.9 (12.9)	Intracranial Thrombectomy, n (%)^a^	
Plaque	70.6 (9.7)	TA	108 (47.6)
Dissection	51.3 (10.7)	SCoT	69 (30.4)
Male gender, n (%)	154 (67.8)	eCAS, n (%)	132 (58.1)
Risk Factors, n (%)		Order of Treatment, n (%)	
Hypertension Diabetes Mellitus Dyslipidemia Smoking Habit	137 (60.4) 36 (15.9) 55 (24.2) 75 (33.0)	Anterograde approach Retrograde approach	67/132 (50.8) 65/132 (49.2)
Baseline NIHSS, median (IQR)		Antiplatelet therapy, n (%)	
All General Anesthesia Conscious Sedation	16.0 (11.0–20.0) 18.0 (13.8–21.3) 15.0 (10.0-18.0)	ASA P2Y12 Inhibitors GpIIb/IIIa Inhibitors	63 (27.8) 8 (3.5) 59 (26.0)
		Timing-minutes, mean (SD)	
IV-FL, n (%)	146 (64.3)	OTG GTR	253.6 (118.4) 76.0 (43.3)

Neuroimaging variables		Outcomes	
Baseline ASPECTS, median (IQR)		90-day mRS ≤2, n (%)^b^	
All *eCAS patients* with SICH without SICH	9.0 (8.0–10.0) 8.0 (7.8–9.0) 9.0 (8.0–10.0)	All *No-SICH patients* with eCAS without eCAS	100 (49.8) 63/96 (65.6) 37/83 (44.6)
Intracranial lesion site, n (%)		mTICI 2b-3, n (%)	175 (77.1)
ICA ICA T-type M1-MCA M2 or distal-MCA Hemodynamic TLs	15 (6.6) 42 (18.5) 97 (42.7) 41 (18.1) 32 (14.1)	Extracranial ICA Recanalization, n (%)^c^	197 (86.8)
		Complications, n (%)	
		All TA SCoT	38 (16.7) 17/108 (15.7) 21/69 (30.4)
Extracranial lesion		SICH, n (%)	22 (9.7)
*1. Etiology*, n (%)		90-day mortality, n (%)^d^	
Plaque Dissection	172 (75.8) 55 (24.2)	All Stroke-related	29 (14.4) 15 (7.5)
*2. Type, n (%)*			
Occlusion Subocclusion or Stenosis	130 (57.3) 97 (42.7)		
Collateral Flow, n (%)			
Good Moderate Poor	99 (43.6) 75 (33.0) 53 (23.4)		

^a^Thrombectomy not performed in 50 patients

^b^90-day mRS available for 201 patients

^c^Extracranial ICA recanalization obtained through percutaneous transluminal angioplasty without eCAS in 65 patients

^d^90-day mortality available for 201 patients

*NIHSS* National Institutes of Health Stroke Scale; *ASPECTS* Alberta Stroke Program Early CT Score; *IV-FL* Intravenous Fibrinolysis; *eCAS* emergent Carotid Artery Stenting; *SICH* Symptomatic Intracerebral Haemorrhage; *ICA* Internal Carotid Artery; *MCA* Middle Cerebral Artery; *TLs* Tandem Lesions; *TA* Thromboaspiration Alone; *SCoT* Stent-retriever Combined Thrombectomy; *ASA* Acetylsalicylic Acid; *GpIIb/IIIa* *Glycoprotein IIb/IIIa* OTG: Onset To Groin puncture; *GTR* Groin Puncture To Reperfusion.

**Table 2:** Univariate analysis and multivariate predictors for 90-day functional independence (mRS ≤ 2)

Variable	mRS ≤ 2 (n=100)	mRS > 2 (n=101)	*p*-*value*	*p-value *(Wald’s test)	OR (95% CI)
Age-years, mean (SD)	62.5 (12.8)	69.5 (12.3)	0.0001	< 0.0001	0.94 (0.91–0.97)
Gender, n (%)					
Male Female	74 (54.0) 26 (40.6)	63 (46.0) 38 (59.4)	0.077	0.020	2.46 (1.15–5.26)
Baseline NIHSS, median (IQR)	15.0 (10.0–18.0)	18.0 (15.0–22.0)	< 0.00001	0.008	0.93 (0.88–0.98)
IV-FL, n (%)					
Yes No	64 (48.5) 36 (52.2)	68 (51.5) 33 (47.8)	0.656		
Baseline ASPECTS, median (IQR)	9.0 (8.0–10.0)	8.0 (7.0–9.0)	0.0002	< 0.0001	1.65 (1.33–2.06)
Intracranial Lesion site, n (%)					
ICA ICA T-type M1 M2 or distal Hemodynamic TLs	5 (38.5) 13 (32.5) 45 (51.1) 22 (62.9) 15 (60.0)	8 (61.5) 27 (67.5) 43 (48.9) 13 (37.1) 10 (40.0)	0.062		
Extracranial Lesion					
*1. Etiology*, n (%)					
Plaque	69 (45.1)	84 (54.9)	0.021		
Dissection	31 (64.6)	17 (35.4)			
*2. Type*, n (%)					
Occlusion	59 (50.4)	58 (49.6)	0.886		
Subocclusion or Stenosis	41 (48.8)	43 (51.2)			
Collateral Flow, n (%)					
Good	56 (62.9)	33 (37.1)	< 0.00001	0.013	0.57 (0.37–0.87)
Moderate	34 (50.7)	33 (49.3)			
Poor	10 (22.2)	35 (77.8)			
Anesthesia, n (%)					
General Anesthesia Conscious Sedation	42 (41.2) 58 (58.6)	60 (58.8) 41 (41.4)	0.017		
Intracranial Thrombectomy, n (%)					
TA	52 (51.5)	49 (48.5)	0.141		
SCoT	23 (38.3)	37 (61.7)			
eCAS, n (%)					
Yes No	63 (54.3) 37 (43.5)	53 (45.7) 48 (56.5)	0.154		
Antiplatelet Therapy					
*1. All*, n (%)					
Yes No	61 (53.5) 39 (44.8)	53 (46.5) 48 (55.2)	0.256		
*2. Class*, n (%)					
ASA P2Y12 Inhibitors GpIIb/IIIa Inhibitors	27 (48.2) 3 (100) 31 (56.4)	29 (51.8) 0 (0) 24 (43.6)	0.237		
Order of treatment, n (%)					
Anterograde approach Retrograde approach	32 (54.2) 31 (54.4)	27 (45.8) 26 (45.6)	1		
Timing-minutes, mean (SD)					
OTG GTR	250.1 (121.0) 70.3 (43.3)	257.9 (117.9) 84.7 (42.8)	0.652 0.022		
Complications, n (%)					
No Yes	90 (52.9) 10 (32.3)	80 (47.1) 21 (67.7)	0.049	0.009	0.27 (0.10–0.72)
mTICI, n (%)					
2b-3 0-2a	88 (56.4) 12 (26.7)	68 (43.6) 33 (73.3)	0.0006		
Extracranial ICA Recanalization, n (%)					
Yes No	93 (53.5) 7 (25.9)	81 (46.5) 20 (74.1)	0.012	0.001	6.05 (2.03– 18.06)
SICH, n (%)					
Yes No	0 (0) 100 (55.9)	22 (100) 79 (44.1)	< 0.00001

**Table 3:** Univariate analysis for technical outcomes (mTICI 2b-3 and Extracranial ICA Recanalization)

Variable	mTICI 0-2a (n=52)	mTICI 2b-3 (n=175)	*p-*value
Age-years, mean (SD)	65.5 (11.3)	66.1 (13.4)	0.748
Gender, n (%)			
Male Female	36 (23.4) 16 (21.9)	118 (76.2) 57 (78.1)	0.867
Baseline NIHSS, median (IQR)	16.0 (12.0–20.8)	16.0 (11.0–20.0)	0.719
IV-FL, n (%)			
Yes No	32 (21.9) 20 (24.7)	114 (78.1) 61 (75.3)	0.625
Baseline ASPECTS, median (IQR)	8.5 (7.8–9.0)	9.0 (8.0–10.0)	0.114
Intracranial Lesion site, n (%)			
ICA ICA T-type M1 M2 or distal Hemodynamic TLs	4 (26.7) 12 (28.6) 16 (16.5) 12 (29.3) 8 (25.0)	11 (73.3) 30 (71.4) 81 (83.5) 29 (70.7) 24 (75.0)	0.332
Extracranial Lesion			
*1. Etiology*, n (%)			
Plaque Dissection	37 (21.5) 15 (27.3)	135 (78.5) 40 (72.7)	0.718
*2. Type*, n (%)			
Occlusion Subocclusion or Stenosis	35 (26.9) 17 (17.5)	95 (73.1) 80 (82.5)	0.111
Collateral Flow, n (%)			
Good	21 (21.2)	78 (78.8)	0.361
Moderate	15 (20.0)	60 (80.0)	
Poor	16 (30.2)	37 (69.8)	
Anesthesia, n (%)			
General Anesthesia Conscious Sedation	26 (25.0) 26 (21.1)	78 (75.0) 97 (78.9)	0.528
Intracranial Thrombectomy, n (%)			
TA	21 (19.4)	87 (80.6)	0.574
SCoT	16 (23.2)	53 (76.8)	
eCAS, n (%)			
Yes No	18 (13.6) 34 (35.8)	114 (86.4) 61 (64.2)	0.0001
Order of treatment, n (%)			
Anterograde approach Retrograde approach	8 (11.9) 10 (15.4)	59 (88.1) 55 (84.6)	0.619
Timing-minutes, mean (SD)			
OTG GTR	261.5 (105.0) 87.0 (42.3)	251.3 (122.1) 73.3 (43.2)	0.572 0.064
Complications, n (%)			
Yes No	14 (36.8) 38 (20.1)	24 (63.2) 151 (79.9)	0.034
Extracranial ICA Recanalization, n (%)			
Yes No	30 (15.2) 22 (73.3)	167 (84.8) 8 (26.7)	< 0.00001

Variable	No Extracranial ICA Recanalization (n=30)	Extracranial ICA Recanalization (n=197)	*p-*value
Age-years, mean (SD)	65.8 (13.4)	65.8 (12.8)	0.998
Gender, n (%)			
Male Female	20 (13.0) 10 (13.7)	134 (87.0) 63 (86.3)	1
Baseline NIHSS, median (IQR)	15.5 (10.8-21.0)	16.0 (11.0-20.0)	0.803
IV-FL, n (%)			
Yes No	16 (11.0) 14 (17.3)	130 (89.0) 67 (82.7)	0.220
Extracranial Lesion			
*1. Etiology*, n (%)			
Plaque Dissection	20 (11.6) 10 (18.2)	152 (88.4) 45 (81.8)	0.252
*2. Type*, n (%)			
Occlusion Subocclusion or Stenosis	23 (17.7) 7 (7.2)	107 (82.3) 90 (92.8)	0.028
eCAS, n (%)			
Yes No	6 (4.5) 24 (25.3)	126 (95.5) 71 (74.7)	<0.00001
Antiplatelet Therapy			
*1. All*, n (%)			
Yes No	10 (7.7) 20 (20.6)	120 (92.3) 77 (79.4)	0.006
*2. Class*, n (%)			
ASA P2Y12 Inhibitors GpIIb/IIIa Inhibitors	6 (9.5) 0 (0) 4 (6.8)	57 (90.5) 8 (100) 55 (93.2)	0.810
1 Order of treatment, n (%)			
Anterograde approach Retrograde approach	4 (6.0) 2 (3.1)	63 (94.0) 63 (96.9)	1
Timing-minutes, mean (SD)			
OTG GTR	223.7 (89.4) 86.4 (36.1)	257.7 (121.4) 74.9 (43.9)	0.091 0.198
Complications, n (%)			
Yes No	2 (5.3) 28 (14.8)	36 (94.7) 161 (85.2)	0.186

**Table 4:** Univariate analysis and multivariate predictors for SICH

Variable	No SICH (n = 205)	Yes SICH (n = 22)	*p*-*value*	*p*-value (Wald’s test)	OR (95% CI)
Age-years, mean (SD)	65.7 (13.2)	68.0 (10.6)	0.366		
Gender, n (%)					
Male Female	140 (90.9) 65 (89.0)	14 (9.1) 8 (11.0)	0.639		
Baseline NIHSS, median (IQR)	16.0 (11.0–20.0)	18.0 (10.0–20.0)	0.795		
IV-FL, n (%)					
Yes No	130 (89.0) 75 (92.6)	16 (11.0) 6 (7.4)	0.486		
Baseline ASPECTS, median (IQR)	9.0 (8.0–10.0)	8.0 (7.3–9.0)	0.026	< 0.0001	0.60 (0.51–0.70)
Intracranial Lesion site, n (%)					
ICA ICA T-type M1 M2 or distal Hemodynamic TLs	13 (86.7) 37 (88.1) 83 (85.6) 40 (97.6) 32 (100)	2 (13.3) 5 (11.9) 14 (14.4) 1 (2.4) 0 (0)	0.035		
Extracranial Lesion					
*1. Etiology*, n (%)					
Plaque Dissection	153 (89.0) 52 (94.5)	19 (11.0) 3 (5.5)	0.299		
*2. Type*, n (%)					
Occlusion Subocclusion or Stenosis	117 (90.0) 88 (90.7)	13 (10.0) 9 (9.3)	1		
Collateral Flow, n (%)					
Good Moderate Poor	93 (93.9) 68 (90.7) 44 (83.0)	6 (6.1) 7 (9.3) 9 (17.0)	0.113		
Anesthesia, n (%)					
General Anesthesia Conscious Sedation	94 (90.4) 111 (90.2)	10 (9.6) 12 (9.8)	1		
Intracranial Thrombectomy, n (%)					
TA SCoT	98 (90.7) 57 (82.6)	10 (9.3) 12 (17.4)	0.160		
eCAS, n (%)					
Yes No	112 (84.8) 93 (97.9)	20 (15.2) 2 (2.1)	0.001	0.002	8.18 (2.15-31.12)
Antiplatelet Therapy					
*1. All*, n (%)					
Yes No	113 (86.9)	17 (13.1)	0.067		
*2. Class*, n (%)					
ASA P2Y12 Inhibitors GpIIb/IIIa Inhibitors	54 (85.7) 8 (100) 51 (86.4)	9 (14.3) 0 (0) 8 (13.6)	0.736		
Order of treatment, n (%)					
Anterograde approach Retrograde approach	59 (88.1) 53 (81.5)	8 (11.9) 12 (18.5)	0.338		
Timing-minutes, mean (SD)					
OTG GTR	250.3 (112.7) 74.4 (43.1)	281.8 (160.3) 89.8 (43.8)	0.379 0.131		
Complications, n (%)					
Yes No	28 (73.7) 177 (93.7)	10 (26.3) 12 (6.3)	0.0008	< 0.0001	6.27 (2.24–17.54)
mTICI, n (%)					
2b-3 0-2a	158 (90.3) 47 (90.4)	17 (9.7) 5 (9.6)	1		
Extracranial ICA Recanalization, n (%)					
Yes No	176 (89.3) 29 (96.7)	21 (10.7) 1 (3.3)	0.324		

The functional independence was achieved in 100/201 (49.8%) cases. Patients who performed EVT under general anesthesia showed lower functional independence rates (58.8% vs 41.4%; *p* = 0.017) and higher median baseline NIHSS (median, 18.0; IQR, 13.8–21.3 vs. median, 15.0; IQR, 10.0–18.0; *p* = 0.0002) compared to conscious sedations.

A 2b-3 mTICI score was observed in 175/227 (77.1%) cases. Mechanical thrombectomy was not performed in 50/227 (22.0%) patients: IVO was more distal than M3-MCA division in three cases, extracranial stenosis was too tight to be crossed in 15 and a “hemodynamic TL” was present in 32.

Among the 146 patients receiving both IV-FL and EVT, no statistical differences in terms of good cerebral reperfusion (78.1% vs. 75.3%; *p* = 0.625) and of functional independence (51.5% vs. 47.8%; *p* = 0.656) were found compared to the 81 cases not undergoing IV-FL.

A carotid stent was positioned in 132/227 (58.1%) cases. Extracranial ICA recanalization was obtained in 197/227 (86.8%) patients, with 126 of them undergoing eCAS (therefore, in 6/132 eCAS patients extracranial ICA was not recanalized). For the other patients, recanalization was achieved through percutaneous transluminal angioplasty alone. Furthermore, extracranial recanalization was achieved in 27/32 “hemodynamic TLs” (in 23 patients through eCAS while via angioplasty in 4).

Among the 132/227 (58.1%) eCAS patients (105/172 atheromasic plaques, 61.0% vs. 27/55 dissections, 49.1%; *p* = 0.157), in 67 (50.8%) cases the stent was positioned with an anterograde approach (23 “hemodynamic TLs”), while in 65 (49.2%) via a retrograde one.

With regard to the etiopathogenesis of the extracranial lesion, atheromasic patients were older compared to those with dissection (mean, 70.6 years; SD, 9.7 vs. mean, 51.3 years; SD, 10.7; *p* < 0.00001). However, independently from the etiology, both plaque and dissection patients achieved similar extracranial recanalization rates (88.4% vs. 81.8%, *p* = 0.252).

ECAS was significantly associated with extracranial recanalization (95.5% vs. 74.7%; *p* < 0.00001), good cerebral reperfusion (86.4% vs. 64.2%; *p* = 0.0001) and higher SICH rates (15.2% vs 2.1%; *p* = 0.001) but not with 90-day functional improvement (54.3% vs. 43.5%; *p* = 0.154).

However, considering only patients without SICH, those who underwent eCAS achieved 90-day functional independence more frequently (65.6% vs. 44.6%; *p* = 0.007).

Furthermore, among the 132 patients undergoing eCAS, the median baseline ASPECTS was significantly lower in the 20 ones who developed SICH (median, 8.0; IQR, 7.8–9.0 vs. median, 9.0; IQR, 8.0–10.0; *p* = 0.009).

Super selective antiplatelet therapy was administered in 130/227 (57.3%) procedures, in particular ASA in 63 (27.8%), P2Y12 inhibitor in 8 (3.5%) and GpIIb/IIIa inhibitor in 59 (26.0%): 55, 8 and 56 of these patients, respectively, underwent eCAS.

Procedural complications occurred in 38/227 (16.7%, 10.1% embolization of new territories and 6.6% iatrogenic perforation or dissection), more frequently when using a stent-retriever (15.7% vs. 30.4%; *p* = 0.025). SICH was observed in 22/227 (9.7%) patients, none of them having a “hemodynamic TL.” Rate of 90-day mortality was 29/201 (14.4%), with 15 cases of stroke-related death AIS (14 of them from SICH).

With reference to the timings recorded, no statistical association was found between OTG and the outcomes considered. On the contrary, patients achieving 90-day functional independence showed shorter GTR (mean, 70.3; SD, 43.3 vs. mean, 84.7; SD, 42.8; *p* = 0.022).

At the multivariate analysis, the strongest predictors of good clinical outcome were: young age (mean, 62.5; SD, 12.8 vs. mean, 69.5; SD, 12.3; OR, 0.94; 95% CI, 0.91–0.97; *p* > 0.0001), low median baseline NIHSS (median, 15.0; IQR, 10.0–18.0 vs. median, 18.0; IQR, 15.0–22.0; OR, 0.93; 95% CI, 0.88–0.98; *p* = 0.008), high baseline ASPECTS (median, 9.0; IQR, 8.0–10.0 vs. median, 8.0; IQR, 7.0–9.0; OR, 1.65; 95% CI, 1.33–2.06; *p* < 0.0001), extracranial ICA recanalization (53.5% vs. 46.5%; OR, 6.05; 95% CI, 2.03–18.06; *p* = 0.001), good collateral flow (62.9% vs. 37.1%; OR, 0.57; 95% CI, 0.37–0.87; *p* = 0.013) and absence of procedural complications (32.3% vs. 67.7%; OR, 0.27; 95% CI, 0.10–0.72; *p* = 0.009).

Conversely, male gender was strongly associated with poor functional outcome (54% vs. 46%; OR, 2.46; 95% CI, 1.15–5.26; *p* = 0.020).

SICH predictors were: low baseline ASPECTS (median, 9.0; IQR, 8.0–10.0 vs. median, 8.0; IQR, 7.3–9.0; OR, 0.60; 95% CI, 0.51–0.70; *p* < 0.0001), occurrence of complications (73.7% vs. 26.3%; OR, 6.27; 95% CI, 2.24–17.54; *p* < 0.0001) and carotid stent placement (84.8% vs. 15.2%; OR, 8.18; 95% CI, 2.15–31.12; *p* = 0.002).

## Discussion

In our experience, TLs prevalence was 15.3% of all the consecutive stroke cases treated endovascularly in the time frame considered (n = 227/1482). This data is consistent with those of the ESCAPE and REVASCAT trials [2, 5], which reported TLs rates of 12.7% and 15.8%, respectively.

Currently, no recommendations or guidelines regarding TLs management are available [13] and therefore the choice about whether and how to treat is left to the single-center experience.

This paper provides a real-life view on these challenging situations and identifies predictors of good technical and clinical outcome.

Furthermore, it is our opinion that current definition of TL as simultaneous occlusion of the extracranial ICA together with an anterior intracranial large vessel [7–11] presents several limitations. First, it does not stress the causal link between extracranial and intracranial lesions; second, it does not consider small vessels occlusions, although they could cause serious clinical consequences [16]; third, it excludes other injurious mechanisms, different from occlusion, which could cause severe neurological symptoms, as hemodynamic impairment.

For these reasons, according to the authors, a TL should be defined as the “simultaneous presence of two lesions on the same vascular axis, being the extracranial lesion, either an ICA plaque or dissection, the actual cause of the intracranial one, either for thromboembolism or for hemodynamic impairment.”

In the following paragraphs, we will discuss the variables that correlated the most with the outcomes considered.

### Clinical features

At 90-day follow-up, 49.8% of the patients achieved functional independence, similar to what observed by Gory et Al. [17]. The strongest predictors of good clinical outcome were young age, low baseline NIHSS, high baseline ASPECTS, good collateral flow and extracranial ICA recanalization (Table 2), as reported by other studies [18–20].

### Extracranial lesion etiology

The 90-day functional independence rate was significantly lower in patients with carotid plaques compared to those with dissections. Although atherosclerosis might be associated with better clinical outcome, being a slow-growing disease that allows the development of collateral circulation [17], our result could be interpreted by considering the older age of plaque patients, who therefore had a lower functional reserve compared to those with dissection.

Nevertheless, both plaques and dissections achieved similar rates of extracranial recanalization, suggesting that extracranial lesion etiology should not condition the decision of performing EVT, as already suggested by Gory et al. [17]

In addition, no differences were found regarding stent placement between plaque and dissection patients, meaning that the necessity of performing eCAS did not correlate with extracranial lesion etiology.

### Procedural, pharmacological and technical features

Today, no consensus exists about optimal anesthetic strategy during EVT for AIS [21, 22].

In our TLs series, conscious sedation was significantly associated with higher 90-day functional independence rates at univariate analysis: this result is out of line with SIESTA, AnStroke and GOLIATH trials [23–25]. However, patients receiving general anesthesia had higher median baseline NIHSS scores and thus more severe AIS, compared to those receiving conscious sedation.

Furthermore, patients who received IV-FL did not achieve higher rates of good cerebral reperfusion and functional independence, confirming that TLs are poorly responsive to IV-FL, independently from the etiology [12]. This result is in favor with ongoing randomized controlled trials which hypothesize the non-inferiority of mechanical thrombectomy compared to bridging therapy (NCT03192332; ISRCTN80619088; NCT03469206; NCT 03,494,920).

#### Mechanical thrombectomy

Regarding intracranial thrombectomy technique, no significant differences in terms of good cerebral reperfusion (Table 3) and of good clinical outcome (Table 2) were found between TA and SCoT, even if a stent-retriever was used less frequently. These results are in line with recent studies [26, 27]. However, in the present study, the use of a stent-retriever resulted in significantly higher rates of procedural complications.

#### Emergent carotid artery stenting

Today, there are no standardized guidelines regarding eCAS in literature [8].

**Fig. 1:**
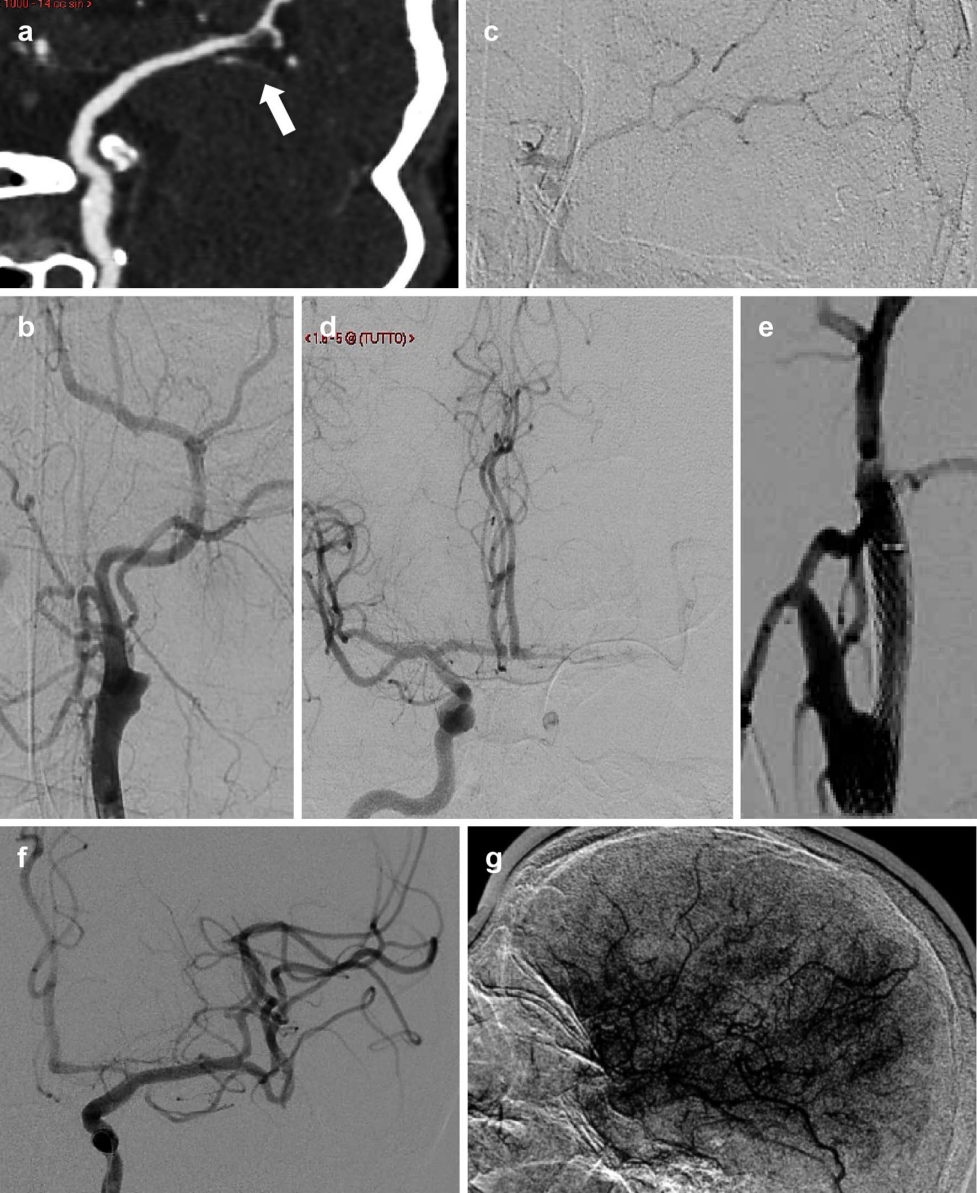
Patient with aphasia, right upper limb hyposthenia and paresthesia (NIHSS 9). Computed tomography angiography, performed at the Spoke Hospital, revealed left M1-MCA sub-occlusion (**a**, arrow). Patient was centralized for thrombectomy to the Hub Hospital immediately after administration of IV-FL. Digital subtraction angiography revealed a complete occlusion of the left extracranial ICA (**b**), with normal opacification of the previously sub-occluded vessel via retrograde anastomotic filling from the ipsilateral ophthalmic artery (**c**). For these reasons, intracranial thrombectomy was not carried out. Since hemodynamic impairment was observed, as poor activation of Willis’ circle (**d**), it was decided to perform eCAS (**e**). Post-procedure angiography showed good cerebral reperfusion (**f**, **g**), mTICI 2b. 90-day mRS 0

In our series, eCAS was significantly associated with extracranial recanalization and good cerebral reperfusion. Therefore, by restoring an adequate anterograde flow, eCAS could be a key factor in achieving a good intracranial reperfusion, especially when hemodynamic impairment occurs (Fig. 1).

Nevertheless, stenting was not significantly associated with 90-day functional improvement, as also reported by Zhu et Al. in their analysis adjusted for confounders [7]. This could depend on the close association between eCAS and SICH: if only patients without SICH are considered, those who underwent eCAS achieved higher 90-day functional independence rates.

### Symptomatic intracerebral hemorrhage (SICH)

In our series, SICH occurred in 9.7% of the total cases, and this is in line with the 10% cutoff value of SICH following EVT, established as a standard of practice by the 2018 international multi-society guidelines [28].

Unfortunately, no patient with SICH achieved 90-day functional independence (Table 2). As expected, SICH was strongly associated with low baseline ASPECTS, procedural complications and carotid stent placement, independently from the antiplatelet drug regimen and from IV-FL administration (Table 4).

ECAS and SICH are generally linked by the so-called “hyperperfusion syndrome,” mostly in atheromasic patients [29, 30]. Interestingly, we found no statistical differences in terms of SICH between plaques and dissections.

Furthermore, considering only patients who underwent eCAS, SICH was more frequently observed in cases of low baseline ASPECTS, pointing out that a careful patient preselection based on the extent of the ischemic core, under strict blood pressure control, might reduce the risk of SICH [31, 32].

Antiplatelet therapy, as well as IV-FL, is usually thought to be associated with higher SICH risk [33, 34]. Although in our series the choice of how, how much and which drug to administer to each patient was left to the single-center operator expertise, both the use of antiplatelet therapy or of IV-FL did not increase SICH risk, as also reported by Zhu et Al. [34]. Therefore, our result is in favor of using antiplatelet drugs and, in accordance with the current literature, preferably with greater, faster and shorter antiplatelet activity [35].

### Limitations and strengths

We are aware that this study has several limitations: first of all, its retrospective, uncontrolled and observational design. Its multicenter design allowed a broader patients recruitment, but it also resulted in higher heterogeneity regarding techniques, devices and patient selection for EVT in each participating center. Furthermore, uniform imaging assessment of stent patency was not evaluated at late follow-up, although pre/post and procedural neuroimaging data were consensually reviewed by two expert readers. Additionally, in our analysis we also included the “hemodynamic TLs,” a group that is often not considered by other authors, despite the same hemodynamic consequences. Further studies considering this particular type of TLs separately and involving a greater number of cases are needed to confirm our results.

However, our analysis has a fundamental strength: as opposite to randomized controlled trials, which consider patients selected under ideal conditions, in our series patients are representative of those normally observed in a real-life setting, not enrolled according to severe inclusive criteria.

## Conclusion

This is the first retrospective real-life case series of TLs patients undergoing EVT which also includes the “hemodynamic TL” (here defined as extracranial ICA occlusion or sub-occlusion being the actual cause of AIS for insufficient Willis compensation). In our series, this group showed no significant differences, in terms of 90-day functional independence and technical success rates, compared to “thromboembolic TLs.” However, no patient with “hemodynamic TLs” developed SICH.

High rates of good cerebral reperfusion (77.1%) and of extracranial recanalization (86.8%) were achieved, with consequent significant improvement in 90-day functional outcome, demonstrating that in these complex situations EVT is technically feasible, effective and safe. ECAS was found to correlate with the risk of SICH, unlike antiplatelet therapy and IV-FL. Anyhow, in our series morbidity and mortality rates did not result higher compared to the current literature.

AIS due to TLs still remain challenging for stroke physicians: more data and new trials are needed to better understand how to optimize the reperfusion strategies in this multifactorial, multidistrict, often dramatic vascular pathology.
